# Content Analysis and Quality Evaluation of Cesarean Delivery–Related Videos on YouTube: Cross-sectional Study

**DOI:** 10.2196/24994

**Published:** 2021-07-30

**Authors:** Kyong-No Lee, Yeon Ji Joo, So Yeon Choi, Sung Taek Park, Keun-Young Lee, Youngmi Kim, Ga-Hyun Son

**Affiliations:** 1 Department of Obstetrics and Gynecology Hallym University Kangnam Sacred Heart Hospital Seoul Republic of Korea; 2 Institute of New Frontier Research College of Medicine, Hallym University Chuncheon Republic of Korea

**Keywords:** cesarean delivery, YouTube, internet, quality of information

## Abstract

**Background:**

YouTube is one of the most popular open-access video-sharing websites, and it is also used to obtain health care information. Cesarean delivery is the most common major surgical intervention in many countries. Videos related to cesarean delivery have also been uploaded to YouTube. However, no study has explored the overall quality of cesarean delivery videos on the platform.

**Objective:**

The objective of this study was to analyze the content and evaluate the quality of the most frequently viewed videos related to cesarean delivery that are accessible on YouTube.

**Methods:**

We searched for a total of 18 terms by combining the 6 terms retrieved from Google AdWords and the 3 terms *c section*, *cesarean section*, and *cesarean delivery*, which are used interchangeably. Videos were sorted by view count, and the 100 videos with the highest view counts were chosen. The number of views, duration, likes and dislikes, content type, and source of each video were recorded. In evaluating the quality of the videos, we referred to a previous study. Additionally, we developed a detailed scoring method that comprehensively evaluates the videos related to cesarean delivery by including the necessary information for each element of the cesarean delivery and whether scientific evidence was presented.

**Results:**

Of the 100 videos analyzed, the most prevalent content (n=28) was videos that contained the actual surgical procedure of a cesarean delivery, and the most common source of cesarean delivery videos was physicians (n=30). Videos directly related to cesarean delivery, such as explanation of the surgery and the actual surgical procedure, were mainly uploaded by medical groups and scored higher than the videos indirectly related to cesarean delivery, which were mainly uploaded by nonmedical groups. In addition, videos directly related to cesarean delivery were more often uploaded earlier in time, with lower like ratios compared to indirect videos.

**Conclusions:**

YouTube is currently not an appropriate source for patients seeking information on cesarean delivery.

## Introduction

Cesarean delivery is the most common surgical intervention in many countries [1]. Based on data from 169 countries, 29.7 million births worldwide were estimated to be by cesarean delivery in 2015, which was almost double the number of births in 2000 (16 million) [2]. Cesarean delivery can be lifesaving for the fetus, mother, or both in certain cases, such as dystocia, placenta previa, or abnormal fetal presentation; however, the rapid increase in the rate of cesarean births without any evidence of associated reduction in maternal or neonatal morbidity or mortality raises concerns that cesarean delivery is being performed for the convenience of the patients or physicians even when it is not required [3]. In recent times, the rate of cesarean delivery due to maternal requests has increased to 8% due to fear of labor pain, anxiety about fetal injury, urinary incontinence, or pelvic floor dysfunction [4]. The fear of litigation among physicians has also played a role in the increase in cesarean delivery rates. Moreover, the autonomy of the patient tends to be a more important consideration in deciding the method of delivery. Therefore, it is important for patients to obtain accurate information about cesarean delivery based on scientific evidence.

In the last decade, social media has emerged as an important source of health care–related information. Altogether, 80% of adults in the United States have used the internet to access health care information [5,6]. Among the web-based resources, YouTube, an open-access video-sharing website, is among the three most popular websites, with more than 4 billion videos viewed daily and more than 500 hours of video content uploaded every minute [7]. YouTube is becoming an increasingly popular platform for users to obtain, share, and discuss health information. In providing information, the social media format has the advantage of possible timely updates; however, social media platforms may contain misleading and inappropriate information because there is a lack of regulation of the content and no peer review process [8-10]. To date, no study has yet evaluated cesarean delivery–related information on YouTube. Therefore, the purpose of this study was to describe and analyze the content of the most-viewed videos of cesarean delivery on YouTube to identify features of cesarean delivery–related videos that were watched by the general public. We also evaluated the quality of the videos related to cesarean delivery on YouTube to determine whether accurate and important information was being delivered.

## Methods

### Retrieval of Cesarean Delivery–Related YouTube Videos

We intended to include representative videos about cesarean delivery that the public could access. The Google keyword search tool Google AdWords [11], a method used by Williams et al [12], was used to identify appropriate search terms that the public would use to explore the term “cesarean section” on YouTube. From the original keyword *cesarean section*, a list of popular terms, such as *38 weeks c section*, *second c section*, *3 months after c section*, *first c section*, *repeat c section*, and *c section complications*, was retrieved. Each term was queried by combining 3 terms, namely, *c section*, *cesarean section*, and *cesarean delivery*, and a total of 18 terms were queried. A YouTube search was conducted on April 2, 2021. As the goal of the search was to identify the videos that the public were watching most frequently, the videos were sorted by view count using the YouTube advanced search options. The criteria for including the videos were as follows: (1) English language used, (2) primary content related to cesarean delivery, and (3) acceptable audiovisual quality. The exclusion criteria were as follows: (1) languages other than English, (2) poor audio or visual clarity, (3) animal videos, and (4) duplicate videos.

**Figure 1:**
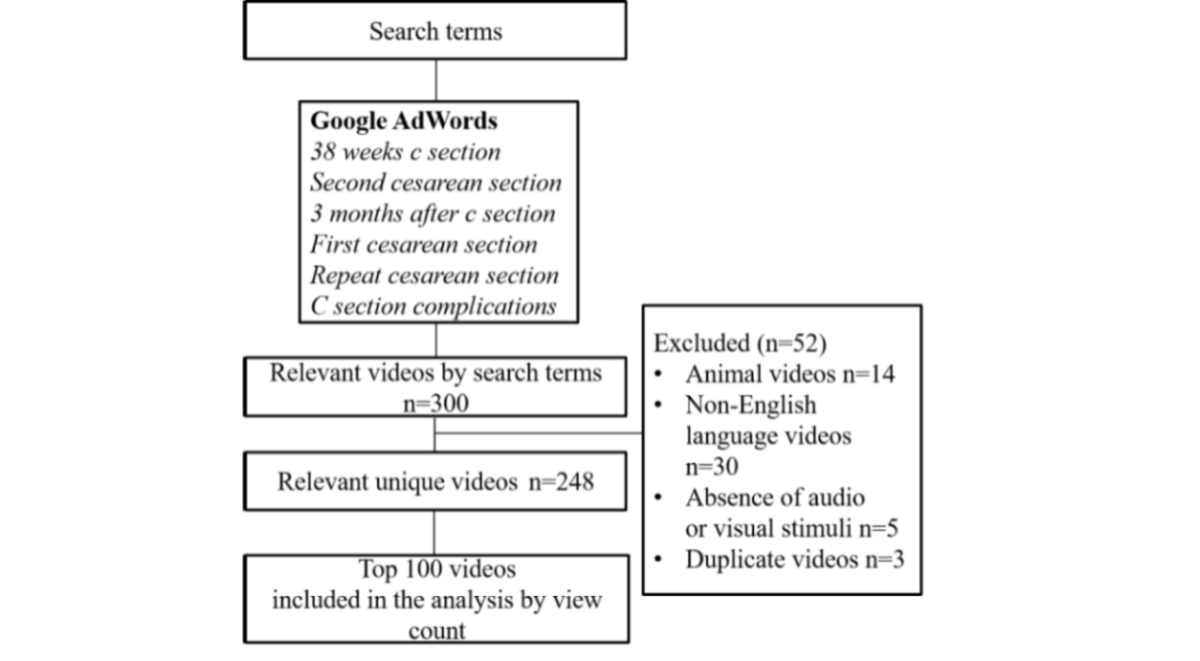
Methodology of selection of YouTube videos for analysis.

For the search terms, the top 300 initial videos were included for review, as determined by the Relevance filter according to YouTube’s algorithm. A total of 52 videos were excluded (14 animal videos, 30 non–English-language videos, 5 videos with poor audio or visual clarity, and 3 duplicate videos). A list of the top 100 videos was populated based on view count, and this list served as the basis for the subsequent analysis. A description of the search strategies is presented in Figure 1.

### Video Assessment

For each video, we collected the objective (video title, number of views, uploaded day, uploader’s name, length of the video, days since upload, total number of “likes” and “dislikes” as depicted by the “thumbs up” and “thumbs down” icons, details and sources related to the cesarean delivery included in the video) and subjective data (the purpose and type of content).

Based on the content in the videos, the videos were categorized into five groups: (1) explanations of surgery (providing general cesarean delivery–related information), (2) surgery procedure (showing or explaining detailed surgical procedure techniques and processes), (3) personal experiences (sharing personal experiences and feelings related to pregnancy and delivery), (4) postpartum care (providing information on postsurgical care, eg, nutrition, exercise, and wound care), and (5) others (mock practice videos in various circumstances, appreciation of medical dramas related to cesarean delivery, and description of surgical instruments). We classified videos that include explanations of surgery and surgical procedures as videos directly related to cesarean delivery, and videos that included personal experiences or postpartum care were classified as videos indirectly related to cesarean delivery (Table 1).

**Table 1:** Characteristics of videos related to cesarean delivery on YouTube (N=100).

Variable				Description		Value, n
**Content**						
	**Directly related**					
		Explanations of surgery	Provide general cesarean delivery–related information		19	
		Surgery procedure	Show or explain detailed surgical procedure techniques and processes		28	
	**Indirectly related**					
		Personal experiences	Share personal experiences and feelings related to pregnancy and delivery		19	
		Postpartum care	Provide information about postsurgical care (eg, nutrition and exercise, wound care)		24	
		Others	Mock practice video in various circumstances, appreciation of medical dramas related to cesarean delivery, and description of surgical instruments		10	
**Source**						
	**Medical**					
		Academic	Authors are affiliated with a university		12	
		Physician	Authors are not affiliated with a university but are physicians		30	
	**Nonmedical**					
		Patient	Woman who has already delivered or is currently pregnant, or her husband		24	
		Commercial	Attention to a product or service		8	
		Paramedical	Allied health therapist, physiotherapist, or dietitian		26	

Based on their authorship, the videos were classified into five basic groups: **(**1) academics (authors were affiliated with a university), (2) physicians (authors were not affiliated with a university but were physicians), (3) patients (a woman who had already delivered or was currently pregnant, or her husband), (4) commercial establishments (attention to a product or service), and (5) paramedical (allied health therapist, physiotherapist, or dietitian). We further categorized the videos uploaded by academic and physician groups into the medical group, and those uploaded by patients, commercial establishments, and paramedical groups were categorized into the nonmedical group (Table 1).

### Quality Assessment

Because there are no established standards for evaluating video quality, we prepared an arbitrary scoring system by referring to a previous study [13-15]. The evaluation factors were divided into the part that evaluates the general quality of the video, whether important information on cesarean delivery was included and explained, and how much scientific evidence was specified (Textbox 1). For general video quality and flow of video contents, each parameter was scored on a scale of 1 to 3. The information on cesarean delivery was divided into 5 elements and scored as follows depending on the degree of explanation: 0 points, not mentioned; 1 point, mentioned briefly; 2 points, mentioned in detail. For videos based on scientific evidence, there were two subdivided items: 0 points were given if there was no mention, and 1 point was given. Thus, the total score of the 5 items ranged from a minimum of 2 to a maximum of 18 points. Three professional obstetricians (two professors at the University Hospital of Obstetrics and one obstetrician fellow) independently evaluated the quality of each video, and the score used for the analysis was the average of the 3 scores.

To assess the popularity of the videos, we used the like ratio (like × 100/[like + dislike]), view ratio (number of views/days), and video power index (VPI) (like ratio × view ratio/100).

**Predetermined list of evaluation factors for the quality of videos on YouTube related to cesarean delivery.**

**General quality (Poor: 1 point; moderate: 2 points; good: 3 points)**

- Overall video quality (audio and video)
- Flow of contents in videos

**Degree to which information is helpful to viewers (Not mentioned: 0 points; mentioned briefly: 1 point; mentioned in detail: 2 points)**

- Indication of cesarean delivery
- Maternal or fetal complications
- Surgical process
- Preoperative preparation, anesthesia
- Postoperative management, postpartum care

**Scientific evidence (No: 0 points; yes: 1 point)**

- Clearly states sources of information
- Provides details of where to obtain additional information on the video topic

### Statistical Analysis

Data are shown as median (range) for continuous variables and as n (%) for categorical variables. Comparisons were made between videos directly related to cesarean delivery and videos with indirectly related contents and between the medical and nonmedical groups using the Mann-Whitney *U* test. Comparisons of the difference in uploaded contents between medical and nonmedical groups were analyzed by the Fisher exact test. The Kruskal-Wallis test with Bonferroni corrections was used to compare scores according to the contents of the uploaded videos. Statistical analysis was conducted using SPSS software, version 27.0 (IBM Corporation). Statistical significance was set at *P*<.05. The reliability between the YouTube videos and the scores by the three obstetricians on the criteria for the items was assessed using intraclass correlation coefficients.

### Ethics Statement

Institutional review board approval was waived for this study because only publicly available data were used.

## Results

The top 100 videos related to cesarean delivery had a collected total of 285,666,251 views (median 1,146,376, range 253,267-31,326,580). The descriptive features of the cesarean delivery–related videos on YouTube are shown in Table 2. The highest view count for a video was 31,326,580. This video was uploaded by a medical media channel in 2008, and it contains general information on cesarean delivery such as operation indication and pelvic anatomy; it also explains detailed surgical processes, preoperative preparation, and postoperative management. This video also received the maximum number of likes (77,164).

**Table 2:** Descriptive features of videos related to cesarean delivery on YouTube (N=100).

Variable	Median (range)
Views, n	1,146,376 (253,267-31,326,580)
Video length (minutes)	5.83 (0.25-34.82)
Time on YouTube (days)	1538.5 (39-5044)
Comments, n	150 (0-5127)
Likes (thumbs up), n	5086 (65-77,164)
Dislikes (thumbs down), n	570 (11-13,534)
Like ratio	88 (60-99)
View ratio	784 (101-73,366)
Video power index	706 (95-66,857)

The median length was 5.83 minutes (range 0.25-34.82), and the majority of videos (80/100, 80%) did not exceed 12 minutes. Videos were uploaded to YouTube approximately 1538.5 days previously on median (range 39-5044), and the most videos were uploaded in 2019. Because the videos included in this study were sorted by number of views, considering that the accumulated views of the recently uploaded videos could be fewer than those uploaded earlier, we observed that the number of uploaded videos began to increase rapidly from 2012 (Table 3).

**Table 3:** Number of videos included in the study (N=100) by year of upload.

Year	Uploaded videos, n
2007	2
2008	1
2009	0
2010	2
2011	2
2012	5
2013	8
2014	9
2015	12
2016	9
2017	14
2018	13
2019	16
2020	6
2021	1

Table 1 shows a description of the categorization according to video contents and authorship. The most prevalent content (n=28) was videos that contained the actual surgical procedure of a cesarean delivery, and in many cases (20/28, 71%), the videos were uploaded by physicians. The second most commonly uploaded videos were videos with information on postpartum care, including postoperative exercises to achieve recovery, nutritional care, and wound care. When the video content was categorized as directly related to cesarean delivery or indirectly related according to the content characteristics of the video, there were 47 directly related videos containing explanations on surgery and actual surgery procedures and 53 indirectly related videos. The source that uploaded the most videos was physicians. When academics and physicians were collectively referred to as a medical group, 42 videos were uploaded by the medical group and 58 videos by the nonmedical group. When analyzing the relationship between the source of the videos and the content, the medical group mainly uploaded videos directly related to cesarean delivery, such as explanations or detailed surgical procedures for cesarean delivery (37/42, 88%), while the nonmedical group mainly uploaded videos about personal experiences and postoperative care (40/58, 69%) (*P*<.001).

To evaluate whether the videos related to cesarean delivery contained accurate and important information on cesarean delivery and whether scientific evidence was presented, we created a detailed scoring method (Textbox 1). The median score was 6 (range 1-16); the video with the highest score was a video containing a well-organized general description of cesarean delivery with the most views, and the video with the lowest score was mainly composed of personal pregnancy photos without including contents related to cesarean delivery. When evaluating the score by each type of content, videos containing personal experience (median score 4, range 2-6) scored significantly lower than videos containing other contents, such as explanations of surgery (median score 9, range 3-16); *P*<.001), surgery procedures (median score 6, range 1-12; *P*<.001) and postpartum care videos (median score 6, range 3-11; *P*=.001). There was a high degree of correlation between the reviewers (intraclass correlation coefficient 0.908, 95% CI 0.872-0.935; *P*<.001).

Next, we created and analyzed the like ratio, view ratio, and VPI to evaluate which videos people were interested in and liked. The video with the highest VPI was a video about the childbirth of an Indian actress, posted by Bollywood Trends, which focuses on birth news rather than cesarean delivery–related information.

We further analyzed how videos related to cesarean delivery uploaded on YouTube differ according to the uploaded content, source, and time when the video was uploaded to YouTube. We compared videos uploaded by the end of 2015 and videos uploaded after 2015, which was the midpoint between 2007 and 2021, and we also compared the videos according to the contents and source.

When we compared videos directly related to cesarean delivery with those indirectly related to cesarean delivery according to the content characteristics of the videos, the videos directly related to cesarean delivery were uploaded earlier (direct vs indirect: median time on YouTube 2247 days, range 266-5044, vs 1298 days, range 39-3821*;* *P*=.02), with a lower like ratio (median 85, range 62-98, vs median 91, range 60-99; *P*=.003) and higher score (median 7, range 1-16, vs median 5, range 2-11; *P*<.001) compared to indirect videos (Table 4). When analyzing the proportion of videos directly related to cesarean delivery compared to all videos, 27/41 videos (66%) directly related to cesarean delivery were uploaded by the end of 2015, but this number decreased to 20/59 (34%) after 2015 (*P*=.009).

**Table 4:** Comparison of the content of the videos (N=100).

Variable	Value, median (range)			*P* value
	Directly related (n=47)	Indirectly related (n=53)		
Views, n	1,616,358 (311,534-31,326,580)	960,159 (253,267-17,553,197)	.23	
Video length (minutes)	5.62 (0.25-32.35)	5.83 (0.95-34.82)	<.99	
Time on YouTube (days)	2247 (266-5044)	1298 (39-3821)	.02	
Comments, n	242 (0-5127)	149 (0-3586)	.78	
Likes (thumbs up), n	4514 (65-77,164)	5217 (195-58,550)	.60	
Dislikes (thumbs down), n	762 (14-13,534)	434 (11-12,521)	.27	
Like ratio	85 (62-98)	91 (60-99)	.003	
View ratio	768 (124-19,265)	786 (101-73,366)	<.99	
Video power index	633 (116-18,128)	730 (95-66,857)	.60	
Score	7 (1-16)	5 (2-11)	<.001	

When analyzed according to source (Table 5), the median number of views and degree of popularity of the videos represented by VPI did not significantly differ between the videos uploaded by the medical group and those by the nonmedical group. However, videos uploaded by the medical group showed significantly higher scores than those by the nonmedical group (median 8, range 4-16, vs median 5, range 1-11; *P*<.001).

When analyzed according to the date when the video was uploaded (Table 6), the videos uploaded after 2015 received more comments (median 271, range 0-5127, vs median 82, range 0-3944; *P*=.005) and had a higher VPI than videos uploaded by the end of 2015 (median 858.5, range 227-66,857, vs 491, range 95-7925; *P*=.005).

**Table 5:** Comparison according to the source of the videos (N=100).

Variable	Value, median (range)			*P* value
	Medical group (n=42)	Nonmedical group (n=58)		
Views, n	1,222,121.5 (281,480-31,326,580)	1,124,691 (253,267-5,286,769)	.84	
Video length (minutes)	8.19 (1.42-32.1)	5.38 (0.25-34.82)	.37	
Time on YouTube (days)	1976.5 (266-5,044)	1494.5 (39-3821)	.31	
Comments, n	256 (0-3944)	148 (0-5127)	.53	
Likes (thumbs up), n	4658.5 (65-77,164)	5087 (195-72,024)	.90	
Dislikes (thumbs down), n	571 (14-8445)	467 (11-13,534)	.90	
Like ratio	86 (62-99)	91 (60-99)	.07	
View ratio	717.5 (124-7949)	795 (101-73,366)	.54	
Video power index	614.5 (116-7796)	740 (95-66,857)	.34	
Score	8 (4-16)	5 (1-11)	<.001	

**Table 6:** Comparison according to the time of upload of the videos to YouTube.

Variable	Value, median (range)			*P* value
	By the end of 2015 (n=41)	After 2015 (n=59)		
Views, n	1,589,552 (253,267-31,326,580)	998,717 (281,480-12,445,056)	.42	
Video length (minutes)	5.62 (0.25-27.32)	7.82 (0.95-34.82)	.68	
Comments, n	82 (0-3944)	271 (0-5127)	.005	
Likes (thumbs up), n	3213 (65-77,164)	6189 (163-72,024)	.049	
Dislikes (thumbs down), n	574 (11-13,534)	488 (14-4518)	.62	
Like ratio	84 (61-98)	91 (60-99)	.004	
View ratio	610 (101-11,254)	1040 (274-73,366)	.004	
Video power index	491 (95-7925)	858.5 (227-66,857)	.005	
Score	6 (1-16)	6 (2-13)	.55	

## Discussion

### Principal Findings

Our study identified that the most viewed video about cesarean delivery was a video that was uploaded in 2008 by Nucleus Medical Media, which is a company that specializes in producing medical illustrations and animations. This video is a well-organized video containing overall information on cesarean delivery, such as operation indication, pelvic anatomy expressed by animations, and detailed step-by-step explanations of surgical procedures, preoperative preparation, and postoperative and postpartum management. This video also received the most likes and the highest score, and it also showed the fifth highest VPI. This video was uploaded in 2008, which is relatively early on YouTube, but it was still considered the best organized and most informative video about cesarean delivery. Of the videos included in this study, 41 videos were uploaded up by the end of 2015, and 59 videos had been uploaded since 2015. Our results showed that videos that were directly related to cesarean delivery were often uploaded at earlier dates, and the proportion of videos that were directly related to cesarean delivery out of all videos after 2015 decreased compared to that of videos uploaded by the end of 2015. In addition, although the difference was statistically insignificant, more than half of the videos from the medical group were uploaded by the end of 2015, while more videos were uploaded by the nonmedical group after 2015 (21/41, 51%, vs 21/59, 36%; *P*=.12). These results suggest that as YouTube becomes more popular and laypeople can easily access and produce content, the number of videos containing contents such as personal experiences and postpartum care being uploaded by laypeople is greater than the number of professional videos containing medical information on cesarean delivery being uploaded by medical groups. However, the videos uploaded after 2015 were more popular, as indicated by their VPIs and like ratios, and they also received more comments than the videos uploaded by the end of 2015. These results suggest that the quality and reliability of information provided by YouTube is not related to popularity. Also noteworthy is that although videos directly related to cesarean delivery had higher quality scores, their like ratios were notably lower than those of videos indirectly related to cesarean delivery; moreover, videos uploaded by medical groups scored higher, but their like ratios tended to be lower. The VPI, which is a comprehensive indicator reflecting popularity, did not show any differences between videos directly related to cesarean delivery and videos indirectly related to cesarean delivery or between videos uploaded by medical and nonmedical groups. These results showed that laypeople expressed their preferences regardless of the quality of the video. These results are similar to those of previous studies. Staunton et al [16] reviewed 50 videos regarding scoliosis and found that videos with greater educational quality were associated with a lower number of views. Ferhatoglu et al [17] recently reported an association between high VPI scores and low Sleeve Gastrectomy Scoring System scores in their review of sleeve gastrectomy videos on YouTube.

**Figure 2:**
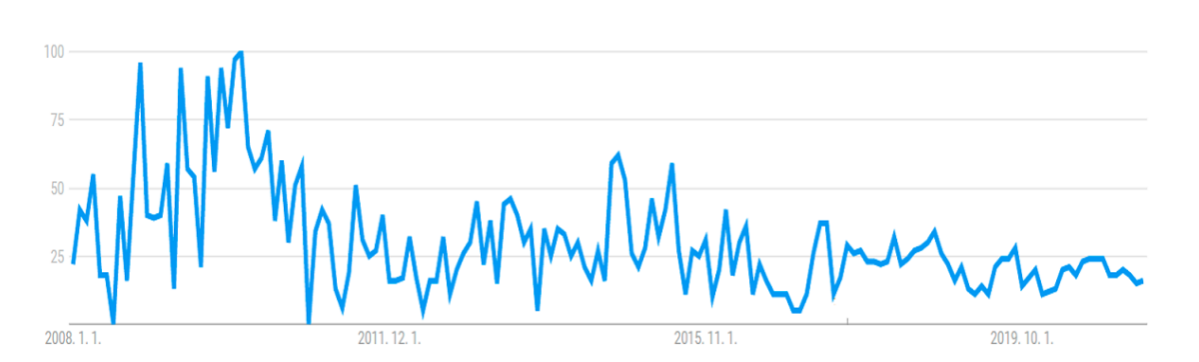
Search trend for the term *cesarean section* on YouTube.

**Figure 3:**
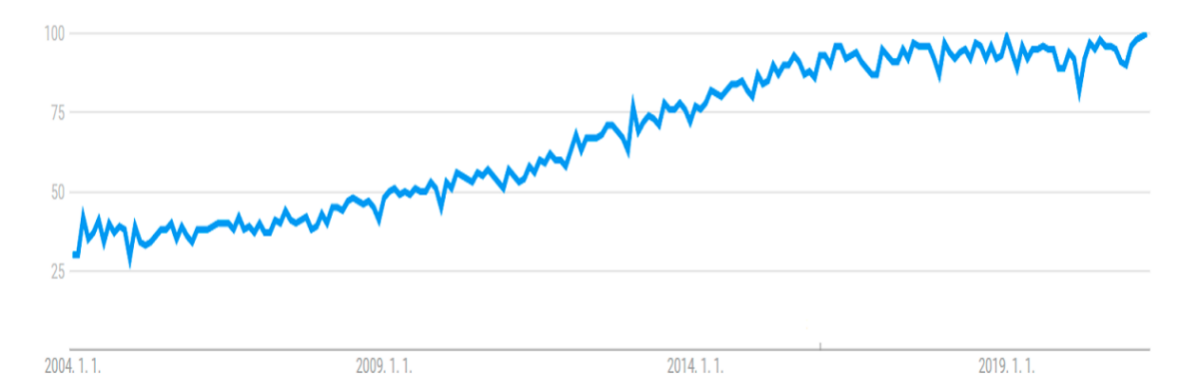
Search trend for the term *cesarean section* on the Google website.

In addition, the results of this study showed that although the total possible score for each video was 18 points, the median score was 6 (range 1-16), which is relatively low. Among the videos, only 13 scored more than 10 points, and the remaining videos showed relatively low scores. The reason for this finding is that most of the videos received low scores in the evaluation items for the information elements related to cesarean delivery (median score 2, range 0-9). Moreover, there were few videos containing the indications for cesarean delivery and maternal or fetal complications that may occur after surgery (n=23 and n=21, respectively), which is important information to be aware of before undergoing a cesarean delivery. In addition, the majority of the videos (80/100, 80%) had a score of 0 in the scientific evidence category, showing that insufficient references were provided for the information in the video. It may not be appropriate to use the scoring method used in this study to evaluate the quality of videos on YouTube, where people can freely produce and upload videos on topics of interest. However, our results showed that videos on YouTube have limitations in providing general and well-organized scientific knowledge of cesarean delivery. It is possible that this limitation is the reason that while the number of searches for “cesarean section” on YouTube has been decreasing over time, the number of YouTube users and searches for “cesarean section” on the Google website has been increasing (Figure 2 and Figure 3). Although YouTube can be seen as a potentially useful medium to search for cesarean delivery–related knowledge and increase awareness, the user must be aware that the information uploaded is not regulated and the quality of the content thus needs to be validated.

### Limitations

This study had some limitations. First, although the assessment method used in this study was adapted from the DISCERN criteria [15], and it reflects the opinions of experts on cesarean delivery, the necessary information for cesarean delivery was included in the evaluation items; however, this method was created arbitrarily by us. Thus, more verification is needed to ensure that the assessment method is suitable for accurately evaluating the quality of videos on cesarean delivery. Second, we only analyzed videos that were in English; thus, sampling bias could have occurred. It is necessary to evaluate videos in other languages for a more comprehensive analysis of the features in videos on cesarean delivery.

### Conclusion

This study is the first to analyze YouTube videos on cesarean delivery, and it contributes to a better understanding of the available information on cesarean delivery that is widely viewed on YouTube. Our results showed that the videos directly related to cesarean delivery, such as explanations of the surgery and actual surgical procedures, were mainly uploaded by medical groups and scored higher than the videos indirectly related to cesarean delivery, which were mainly uploaded by nonmedical groups. In addition, videos directly related to cesarean delivery were more often uploaded earlier in time, and the proportion of videos that were directly related to cesarean delivery decreased after 2015. In our results, when we used the scoring method to evaluate the accuracy of the important information on cesarean delivery, a majority of videos had low scores, showing that YouTube has limitations in delivering accurate information on cesarean delivery.

## Abbreviations

VPI: video power index

## References

[1] Biccard BM, Madiba TE, Kluyts H, Munlemvo DM, Madzimbamuto FD, Basenero A, Gordon CS, Youssouf C, Rakotoarison SR, Gobin V, Samateh AL, Sani CM, Omigbodun AO, Amanor-Boadu SD, Tumukunde JT, Esterhuizen TM, Manach YL, Forget P, Elkhogia AM, Mehyaoui RM, Zoumeno E, Ndayisaba G, Ndasi H, Ndonga AKN, Ngumi ZWW, Patel UP, Ashebir DZ, Antwi-Kusi AAK, Mbwele B, Sama HD, Elfiky M, Fawzy MA, Pearse RM, African Surgical Outcomes Study (ASOS) investigators (2018). Perioperative patient outcomes in the African Surgical Outcomes Study: a 7-day prospective observational cohort study. Lancet.

[2] Boerma T, Ronsmans C, Melesse DY, Barros AJD, Barros FC, Juan L, Moller A, Say L, Hosseinpoor AR, Yi M, de Lyra Rabello Neto D, Temmerman M (2018). Global epidemiology of use of and disparities in caesarean sections. Lancet.

[3] Gregory KD, Jackson S, Korst L, Fridman M (2012). Cesarean versus vaginal delivery: whose risks? Whose benefits?. Am J Perinatol.

[4] Jenabi E, Khazaei S, Bashirian S, Aghababaei S, Matinnia N (2020). Reasons for elective cesarean section on maternal request: a systematic review. J Matern Fetal Neonatal Med.

[5] Atkinson NL, Saperstein SL, Pleis J (2009). Using the internet for health-related activities: findings from a national probability sample. J Med Internet Res.

[6] Finney Rutten LJ, Blake KD, Greenberg-Worisek AJ, Allen SV, Moser RP, Hesse BW (2019). nline health information seeking among US adults: measuring progress toward a Healthy People 2020 objective. Public Health Rep.

[7] Azer SA, Algrain HA, AlKhelaif RA, AlEshaiwi SM (2013). Evaluation of the educational value of YouTube videos about physical examination of the cardiovascular and respiratory systems. J Med Internet Res.

[8] Koller U, Waldstein W, Schatz K, Windhager R (2016). YouTube provides irrelevant information for the diagnosis and treatment of hip arthritis. International Orthopaedics (SICOT).

[9] Cassidy JT, Fitzgerald E, Cassidy ES, Cleary M, Byrne DP, Devitt BM, Baker JF (2017). YouTube provides poor information regarding anterior cruciate ligament injury and reconstruction. Knee Surg Sports Traumatol Arthrosc.

[10] Erdem H, Sisik A (2017). The reliability of bariatric surgery videos in YouTube platform. Obes Surg.

[11] Google Adwords keyword planner.

[12] Williams D, Sullivan SJ, Schneiders AG, Ahmed OH, Lee H, Balasundaram AP, McCrory PR (2014). Big hits on the small screen: an evaluation of concussion-related videos on YouTube. Br J Sports Med.

[13] Hegarty E, Campbell C, Grammatopoulos E, DiBiase AT, Sherriff M, Cobourne MT (2017). YouTube™ as an information resource for orthognathic surgery. J Orthod.

[14] Singh AG, Singh S, Singh PP (2012). YouTube for information on rheumatoid arthritis--a wakeup call?. J Rheumatol.

[15] Charnock D, Shepperd S, Needham G, Gann R (1999). DISCERN: an instrument for judging the quality of written consumer health information on treatment choices. J Epidemiol Community Health.

[16] Staunton PF, Baker JF, Green J, Devitt A (2015). Online curves: a quality analysis of scoliosis videos on YouTube. Spine.

[17] Ferhatoglu MF, Kartal A, Ekici U, Gurkan A (2019). Evaluation of the reliability, utility, and quality of the information in sleeve gastrectomy videos shared on open access video sharing platform YouTube. Obes Surg.

